# Wettability of Two-Dimensional Carbon Allotropes from Molecular Simulations

**DOI:** 10.3390/molecules30153296

**Published:** 2025-08-06

**Authors:** Margaret E. Thornton, Serban G. Zamfir, Dusan Bratko

**Affiliations:** Department of Chemistry, Virginia Commonwealth University, Richmond, VA 23284, USA; meg.thornton9@gmail.com (M.E.T.); zamfirsg@gmail.com (S.G.Z.)

**Keywords:** wetting free energy, contact angle, planar carbon-based materials, Grand Canonical simulations

## Abstract

Force-field Monte Carlo and Molecular Dynamics simulations are used to compare wetting behaviors of model carbon sheets mimicking neat graphene, its saturated derivative, graphane, and related planar allotropes penta-graphene, *γ*-graphyne, and *ψ*-graphene in contact with aqueous droplets or an aqueous film confined between parallel carbon sheets. Atomistic and area-integrated surface/water potentials are found to be essentially equivalent in capturing moderate differences between the wetting free energies of tested substrates. Despite notable differences in mechanical and electric properties of distinct allotropes, the predicted allotrope/water contact angles span a narrow window of weakly hydrophilic values. Contact angles in the range of 80 ± 10° indicate modest hydration repulsion incapable of competing with van der Waals attraction between carbon particles. Poor dispersibility in neat water is hence a common feature of studied materials.

## 1. Introduction

**Figure 1:**
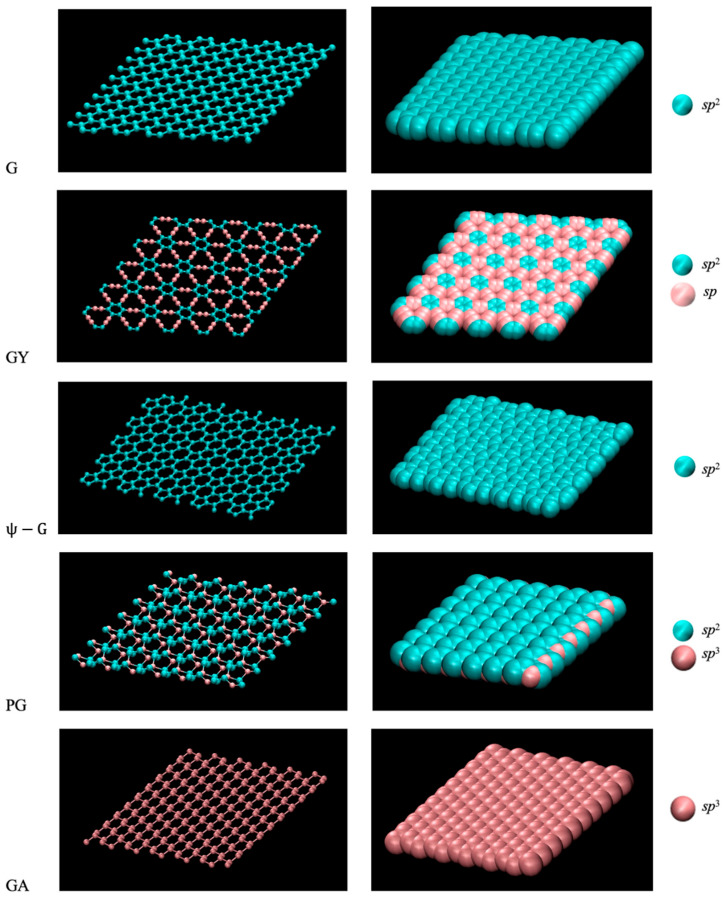
Structures of 241-atom fragment of graphene (G), 300-atom fragments of γ-graphyne (GY) and ψ-graphene (ψ-G), 280-atom fragment of penta-graphene (PG), and 240 carbon atom fragment of graphane (GA) in cpk (**left**) and van der Waals (**right**) VMD 1.9.4 [16] representations. The sizes of atoms shown in the van der Waals representation correspond to Lennard-Jones diameters $\sigma_{CC}^{sp^{n}}$. Fragment/water contact planes are determined by flat layers of exposed atoms.

In addition to explorations of its potential applications, the discovery of graphene [1,2] spurred numerous studies of its derivatives, and search for related two-dimensional allotropes of carbon to increase the range of chemical, mechanical, optical and electric properties of fundamental and pragmatic interest [3,4,5,6,7,8,9]. First principles calculations lead to predictions of multiple planar allotropes of carbon, some of which contain solely *sp*^2^ carbon atoms, e.g., the recently proposed ψ-graphene [10], while others include fractions of *sp*^3^ and/or *sp* carbon atoms [9,11]. A fully saturated analog of graphene, termed graphane, has also been predicted [12] through uniform hydrogenation of carbon atoms. Because of tetrahedral coordination associated with *sp*^3^ hybridization, the presence of *sp*^3^ carbons precludes strict in-plane configurations. As exemplified by graphane and penta-graphene, flat structures can nonetheless be accommodated with minimal corrugations. In graphane, (CH)_n_, *sp*^3^ carbon atoms were predicted [12] and later confirmed by experiment [13] to occupy lattice vertices on parallel planes positioned about 0.25 Å (~14% of atom size) above and below the central plane of the sheet [12,14]. Penta-graphene, a predicted allotrope comprising neat-carbon pentagons [15], on the other hand, contains one third of carbon atoms in *sp*^3^ and two thirds in *sp*^2^ hybridization with the *sp*^3^ ones located at the central plane of the sheet while the *sp*^2^ atoms reside at atomic planes 0.6 Å above and below the central plane. Atomic diameters considerably exceed in-plane interatom distances and only the atoms from the top plane are sterically accessible to water. As can be inferred from Figure 1, the resulting carbon-oxygen contact plane remains essentially flat, analogous to graphene system.

The same observation applies to graphane (bottom panel in Figure 1), another allotrope deviating from the ideal in-plane geometry.

Surface buckling in the presence of tetravalent *sp*^3^ atoms has been found to affect the mechanical rigidity [17], contrasting ideal planar allotropes like graphene, ψ-graphene [10], or graphynes, which contain planar arrangements of *sp*^2^ and *sp* atoms [18,19,20,21,22]. The presence of *sp*^3^ atoms increases the stiffness, while both *sp*^3^ and *sp* components lower the rupture threshold. In cases of GA and GY, in-plane anisotropy of tensile moduli is also predicted [23]. Varied populations of delocalized π electrons associated with connected *sp*^2^ atoms lead to a wide variation in electric properties, from the known metallicity in the case of graphene and predicted metallicity of ψ-graphene, as well as semiconductor properties of γ-graphyne and penta-graphene, to the prediction of insulating behavior of graphane—confirmed following the material’s synthesis in 2009 [13,24]. Apart from graphene and graphane, experimental evidence is scarce and the knowledge about physical properties of planar carbon allotropes commonly relies on predictions from computational models.

Potential applications of new materials often depend on their wetting propensities and possible implications for dispersibility [25] in aqueous media. Literature contains scarce information about wetting properties of the allotropes concerned herein. Out of five systems, only three, graphene [1], graphane [13], and γ-graphyne [26,27] have been synthesized and only graphene has so far been well-characterized in experiments. Because of method differences, impurities, and surface irregularities, reported contact angles of water on suspended graphene span a wide range between 42° and 127° [28,29] but careful decontamination and use of smooth surfaces lead to a likely window between 79° and 85° [30,31,32]. Theoretical studies have been sharply focused on graphene with prevalent outcomes in the range from 80° to 100° [33,34,35]. Molecular dynamics simulations with OPLS force field [36] determined [37] contact angle on graphane at ~73°. The application of reactive force field [38] to penta-graphene resulted [39] in an extreme contact angle of 134° but the same force field predicted just slightly lower contact angle on graphene surface. We are not aware of theoretical results for wetting on ψ-graphene or γ-graphyne. This study employs computer simulations to examine if varied contents of *sp*^2^, *sp*^3^ and *sp* hybridized carbons [19], along with associated structure modifications reflected by notable changes in mechanical and electric properties of 2-D carbon allotropes, can also lead to appreciable differences in their wettabilities. Figure 1 indicates only moderate differences in steric accessibility of different allotropes’ surfaces but the strength of van der Waals interactions with water still varies with hybridization states and densities of carbon atoms. We present a comparison between first-order simulation estimates of wetting free energies and contact angles for a set of prototypical structures ranging from chemically uniform graphene composed of identical *sp*^2^ carbon atoms, to its fully saturated counterpart, graphane where all atoms are in *sp*^3^ state. The computational methodology is benchmarked against existing experimental and theoretical data about graphene systems. In addition to graphene, we concern another pair of ideally planar allotropes, ψ-graphene with *sp*^2^ atoms in alternating pentagon, hexagon and heptagon rings [10], and γ-graphyne [18,21] containing equal shares of *sp*^2^ and *sp* atoms. Lastly, we consider a three-level pentagon-based allotrope penta-graphene with a 2:1 ratio of *sp*^2^ vs. *sp*^3^ carbon atoms. We determine materials’ wetting free energies and water/substrate contact angles using Grand Canonical Monte Carlo (GCMC) [40]/pressure-tensor calculations in nanoconfined water. These calculations employ surface-integrated substrate/water potentials, an approach we validate by comparison with the results of direct contact angle calculations in fully atomistic Molecular Dynamics (MD) simulations [41] for a subset of systems including graphene and both allotropes whose atoms occupy multiple lattice planes, penta-graphene and graphane. We provide methodological specifics in Section 3. In Section 2, we describe our numerical results, which show neat carbon allotrope sheets possess similar wetting affinities irrespective of their structural details. The use of either allotrope, when favored in view of its electric or mechanical requirements, will therefore rarely depend on wetting or dispersibility properties, as they are deemed similar within the entire class. Solvent modifications or carbon functionalization by polar groups may provide viable options when tailored substrate’s wetting and solubility are desired for specific applications.

## 2. Results and Discussion

**Figure 2:**
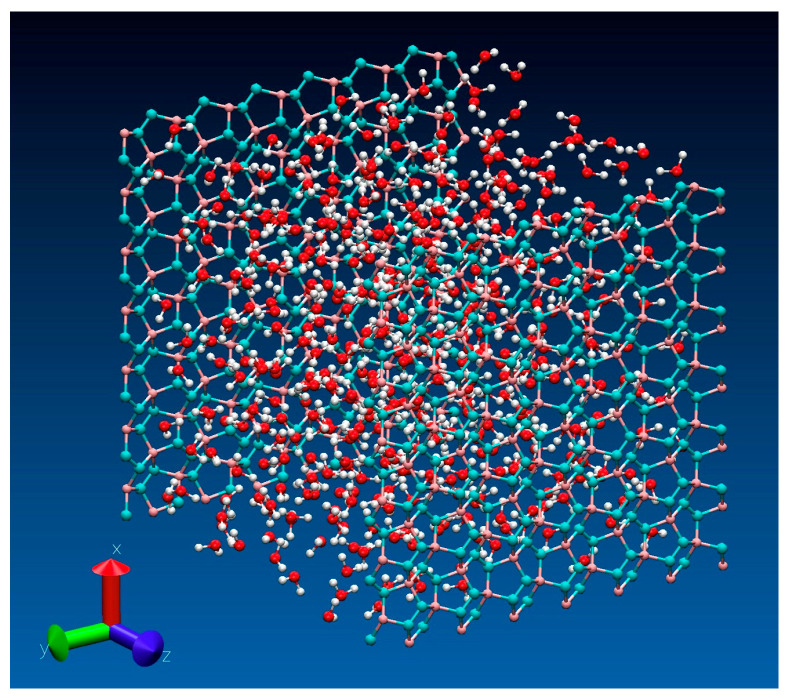
A typical GCMC simulation system with water (O: red, hydrogen: white) between penta-graphene walls (turquoise: *sp*^2^, purple: *sp*^3^ atoms) at separation 25 Å. In case of multilevel walls (two lattice planes occupied by C atoms in graphane, and three planes in penta-graphene), the wall-wall separation corresponds to the distance between the top lattice planes directly exposed to water. The cell is periodically replicated along lateral (*x,y*) directions, with long-range electrostatics captured by 2-D corrected Ewald summation. Water content in the cell is determined through GCMC equilibration with a bulk aqueous phase at ambient conditions. Atom sizes are out of scale to improve visibility.

**Figure 3:**
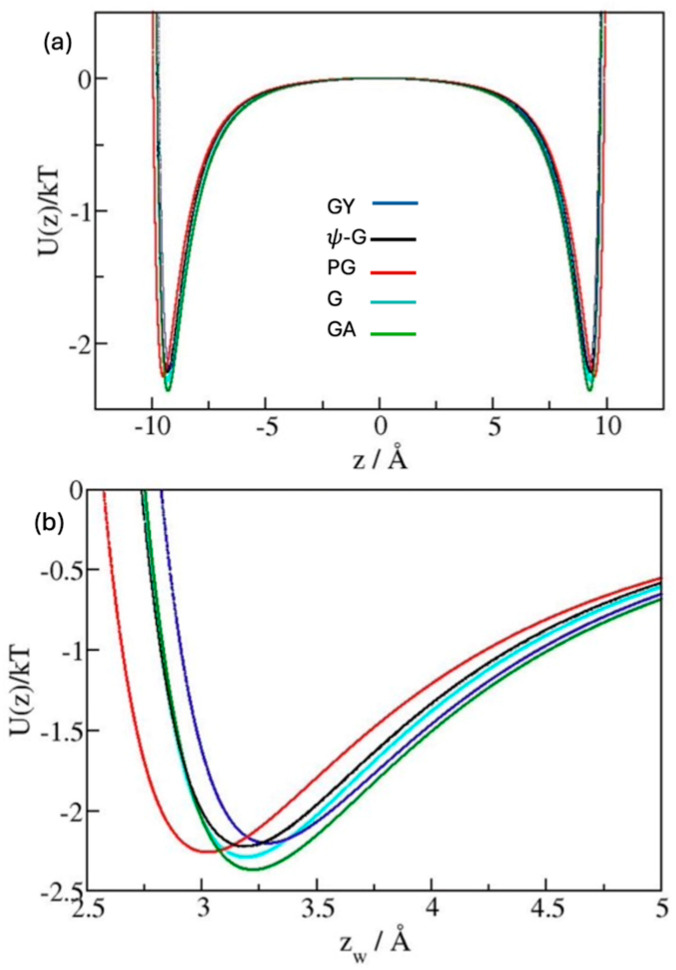
(**a**) Average substrate/water interaction *U*(*z*)/*kT* of water molecules as a function of the normal coordinate (*z*) in the GCMC simulation cell with exposed substrate carbon atoms at *z* = ± 12.5 Å. (**b**) Enlarged area of interest in the proximity of interaction extrema. Here, coordinate z_w_ denotes the distance of water oxygen from the nearest substrate atoms on the left wall, i.e., *z_w_* = z + 12.5 Å. The curves corresponding to graphene (G), ψ-graphene (ψ-G), γ-graphyne (GY), penta-graphene (PG) and graphane (G) reveal relatively small differences in the strengths of net substrate/water attraction.

Approximate predictions of surface wettabilities of graphitic structures can be inferred from the strength of surface/water attraction quantified in terms of the average well-depth of wall-water potential in specified model systems [42,43]. Figure 2 shows our typical simulation setup and Figure 3 compares area-integrated water/wall potentials *U*(*z*)/*kT* for model systems with graphene (G), ψ-graphene (ψ-G), γ-graphyne (GY), penta-graphene (PG), and graphane (GA) confining walls. *U*(*z*) profiles were obtained according to Equations (6) or (7) (Models and Methods) with Lennard-Jones (LJ) parameterizations from Sets 1 and 2 shown in Table 1 and discussed below Equation (2) in the Models and Methods section. To provide a suitable reference, Table 1 also includes new data for three-layer graphite (GT) with identical LJ parameterization for *sp*^2^ carbon atoms. The three-layer model is used since it has been proven [33] representative of graphite with macroscopic dimensions.

**Figure 4:**
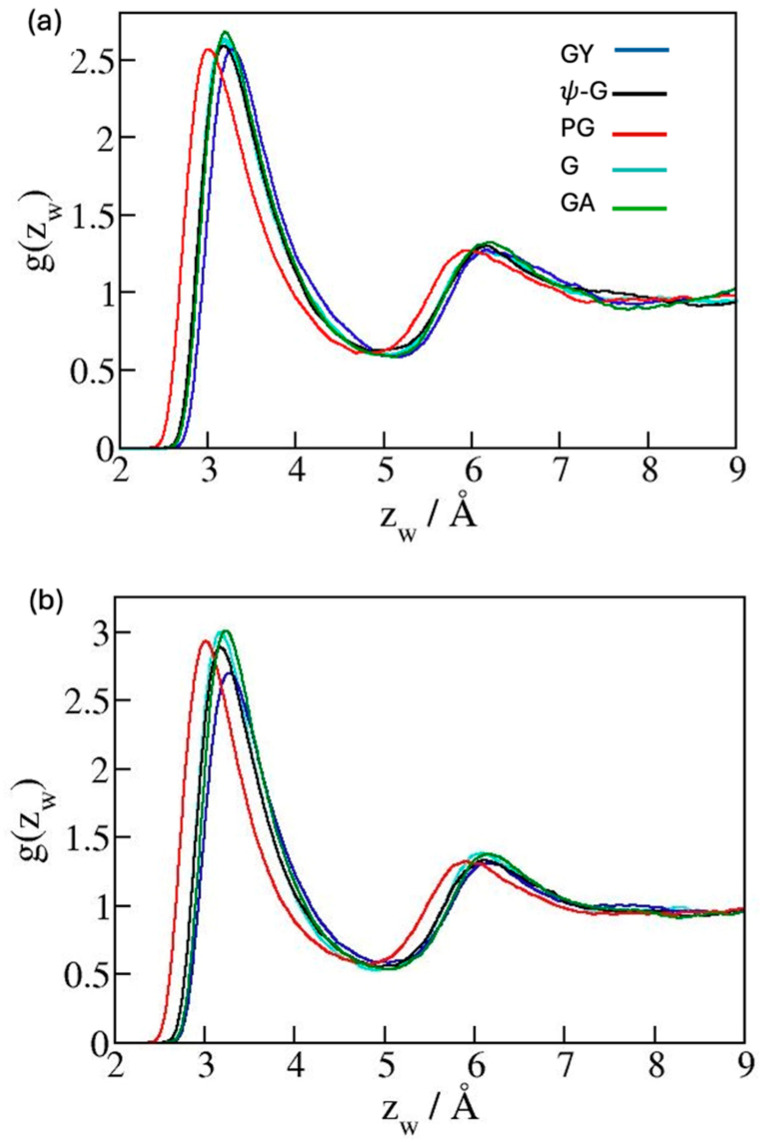
Distribution functions *g*($z_{w})=\frac{\rho_{O}\left(z_{w}\right)}{\rho_{O}^{bulk}}\;$of water oxygen molecules as functions of the distance from the nearest substrate atoms, *z*_w_, at graphene (G), ψ-graphene (ψ-G), γ-graphyne (GY), penta-graphene (PG) and graphane (G) surfaces in with bulk water. $\rho_{O}$ is the density of water oxygens. Graphs (**a**,**b**) use parameterizations shown in Table 1 for Sets 1 and 2, respectively. Peak positions coincide with the minima of water/wall potentials shown in Figure 1. Note the peak heights are not the only predictor of hydration; when compared to graphene, the bigger width of the attractive potential well at the penta-graphene surface also contributes to the somewhat stronger wetting propensity.

The top graph in Figure 3 shows wall/water interaction profiles across the simulation box where a water slab is confined between carbon sheets at separation *h* = 25 Å (See Figure 2), while the bottom graph focuses on the region in the proximity of potential minima. The depth of wall/water potential *U_min_*(z) increases in the order GY, ψ-G, PG, G, and GA as can also be inferred from the inspection of water distribution functions (Figure 4) because the heights of the 1st hydration peaks reflect the relative strengths of substrate/water attraction. Since *U_min_*(*z*) represents an energetic advantage for the formation of wall/water contacts, the above sequence suggests the systems’ wettability (decreasing wetting free energies ∆γ and contact angles θ) to vary in the same order. When comparing substrates with identical surface topologies, the wettability has been shown [34,42,43] to follow this expectation. The analysis described in ref. [34] quantified an almost constant slope of increasing cosθ vs. the strength of interaction between substrate atoms and water oxygens, $\varepsilon_{CO}$. When comparing surfaces with mixed compositions and distinct topologies, *U_min_*(*z*) becomes the measure of combined substrate/water attraction, and an analogous trend is expected to describe the relation between cosθ and *U_min_*(*z*). The wetting propensity of the five allotropes should therefore increase according to the above order, with γ-graphyne featuring the lowest and graphane featuring the highest wettability. The similarity of both the interaction profiles, *U*(*z*_w_), and distribution functions, *g*(*z*_w_), observed with distinct substrates, however, suggests only limited differences in surface wettabilities of the allotropes we are considering.

In Table 1, we quantify surface wettabilities in terms of simulation results for interfacial compressibilities, wetting free energies, and contact angles of water on different substrates. Multiple works demonstrated a direct relation between surface hydrophobicity and local density fluctuations, manifested as excess compressibility of interfacial water [44,45,46,47,48,49]. Column 8 of Table 1 lists liquid compressibilities, κ,

(1) $$\kappa ={\left(\frac{\partial lnN}{\partial P}\right)}_{V,T}=\frac{V\delta N^{2}}{kT{<N>}^{2}}$$

from GCMC simulations of water confined between carbon surfaces of five types shown in Figure 1. Above, *N* is the number of molecules in the confinement volume *V*, *P* is pressure, $\delta N^{2}=<N^{2}>-{<N>}^{2}$, k is Boltzmann constant and *T* temperature. In the next column, we include approximate compressibilities of the hydration layers, $\kappa_{h}$, estimated by presuming the excess compressibility inside the confinement (relative to bulk value $\kappa_{\mathrm{bulk}}^{\mathrm{SPC}/E}=\;$4.6 × 10^−5^ bar^−1^) to originate entirely from the first solvation layer, as has been shown elsewhere [47]. In our systems, the affected fraction of water (molecules located no farther than the position of the 1st density minimum from the nearer wall) was generally within 29.5 ± 1% of the entire aqueous film. The compressibilities increase in the order corresponding to increasing (less negative) wetting free energies and contact angles shown in subsequent columns.

The observed wetting free energies range from ~−5 mJ m^−2^ for GY to −18 mJ m^−2^ for GA, an almost fivefold change; however, the absolute difference of ~13 mJ m^−2^ does not translate to significantly different contact angles (86° vs. 74°) because it remains small in comparison to the surface tension of water.

**Figure 5:**
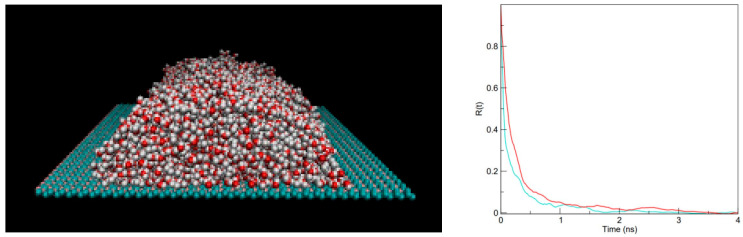
(**left**) A snapshot from Molecular Dynamics simulation of a cylindrical nanodrop consisting of 6591 SPC/E water molecules on a semi-infinite penta-graphene sheet. Atom sizes (red spheres: oxygen, white: hydrogen, turquoise: *sp*^2^ and purple *sp*^3^ carbon, are out of scale. (**Right**) correlation functions $R\left(t\right)=\frac{h\left(t\right)-h\left(\infty \right)}{h\left(0\right)-h\left(\infty \right)}$, where *h*(*t*) is the height of the drop’s center of mass at time *t*. Red and cyan curves *R*(*t*) describe penta-graphene and graphene systems, respectively. Simulations started from a rectangular drop allowed to relax to its average shape with typical relaxation time $\tau =\int_{0}^{\infty }R\left(t\right)dt\;\approx$ 165 ps, considerably longer than 120 ps on neat graphene. Contact angle was determined from the final 6 ns out of 8 ns simulation runs using the drop contour at heights above the bottom two solvation layers.

The final column of Table 1 lists contact angles of four systems obtained directly from the geometries of 6–7 × 10^3^ molecule cylindrical nanodrops on penta-graphene, graphane, and graphene surfaces in MD simulations where we used full-atom substrate representations. Figure 5 shows an MD simulation snapshot for the penta-graphene system with a drop containing 6591 SPC/E water molecules on 120 Å × 240 Å surface and Set 2 LJ parameters from this study. MD results for graphene [34,35] and graphane [37] are from previous works. We include these data to ascertain the reliability of the coarse-grained substrate representation (Equations (6) and (7)) employed in the GCMC calculations. The GCMC and MD contact angles of test systems have a mean difference of 1°, and statistically insignificant mean absolute difference of 2.3°, affirming the reliability of coarse-grained potentials in wettability calculations. Faster droplets’ relaxation rates on graphene compared to penta-graphene surfaces (Figure 5) are indicative of a slightly smoother surface of the former material.

**Figure 6:**
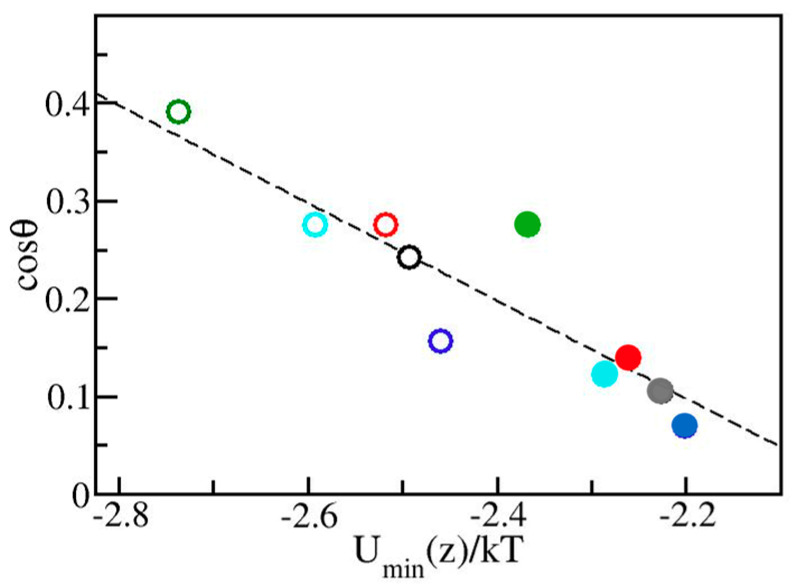
Variation in cosθ with the strength of water/surface attraction quantified in terms of the depth of potential *U_min_*(*z*) for the allotropes listed in Table 1. Colors conform to Figure 3 and Figure 4. Solid and open symbols denote parameterizations from Set 1 and 2, respectively. The dashed line represents a linear fit of the data, including the known point *U_min_*(*z*) = 0, cosθ = −1.

Based on comparisons between graphene models with different force-field parameterizations, earlier works identified water/substrate binding energy as a reliable predictor of contact angle [34,42,43]. To verify if the relation can be generalized to substrates with different surface topologies, in Figure 6 we show the dependence of cosθ on *U_min_*(*z*) for the allotropes considered in Table 1. cosθ is monitored assuming the approximate proportionality of the adhesion strength γ_lv_(1 + cosθ) to *U_min_*(*z*). While the overall trend and the average slope agree with previous work [34], deviations from smooth dependence provide a measure of substrate-specific structural effects. The depth of substrate/water potential remains the main determinant of surface wettability. The calibration provided in Figure 6 enables reasonable first estimates of contact angles for given model solely from the information about *U_min_*(*z*). Judging from the results for our prototypical systems, this approach enables predictions of contact angles with uncertainty of up to ±5 degrees and typical deviation of no more than ± 1–2 degrees, which is close to the statistical noise of the simulation. As an illustrative example, we can consider graphdiyne [23], a single-plane allotrope with low density of C atoms$\;\varrho_{c}≅0.24\;{Å}^{-2}$; a third of the atoms is in *sp*^2^ and two thirds in *sp* state. Using Lennard-Jones parameters from Set 1 in Table 1, Equation (7) yields a *U_min_*(*z*) of −1.96 *kT*, which translates to cosθ≅−0.04 and a model estimate of contact angle at 92° within a confidence window of ~$\pm 5^{{}^{\circ}}$ without performing actual computations.

On the whole, the present results suggest the distinctions among neat carbon allotropes are not sufficient to produce significantly different wettabilities in water. In the literature, only wetting on graphene has so far been characterized in multiple simulation studies, some of which we refer to in the Methods Section. A single work comparing contact angles on graphene and penta-graphene showed rather small differences between the two surfaces [39]; however, weaker carbon-water attraction resulting from the force field [38] used therein resulted in considerably higher contact angles in comparison to those obtained in the present work.

According to our simulation results, free energies of all surfaces considered in Table 1 vary within a window of no more than 20 mJ m^−2^. Given the typical association free energies between graphitic surfaces in water are in the neighborhood of −2 × 10^2^ mJ m^−2^ [50,51], small wettability differences between the known or emerging carbon allotropes cannot significantly improve the material’s dispersibility in water. Except for smallest (oligomer) fragments, aqueous dispersions of carbon allotropes can therefore only be sustained through chemical modifications like addition of surfactant [52,53] or decoration by polar functionalities [54]. Plausible solubilization mechanisms can also arise from asymmetric adsorption of salt ions, with deviations from regional neutrality potentially enhanced [55] by thermal fluctuations. These efforts can be eased by higher original wettability of neat systems.

While favored due to their computational efficiency, the simplified force fields we use cannot directly address possible roles of intramolecular degrees of freedom and quantum mechanical effects. As such, the present approach provides first order estimates and mostly qualitative conclusions. Its reliance on empirical potentials reproducing wetting behaviors of known systems like graphite, graphene and diamond surfaces is likely to offset some of the model approximations through parameterization. As an example, in a separate study [35], we showed the inclusion of graphene polarization to strengthen close range graphene/water attraction by about 3%, a secondary effect commonly absorbed in empirical parameterization of a nonpolarizable model. For the purposes of this study, the accuracy of the latter approach clearly depends on the transferability of effective interatomic potentials for the three hybridization states. While methodological advances are hoped to preserve qualitative findings from our work, a definitive answer, as well as extensions to more complex systems discussed in the preceding paragraph, will necessitate electronic structure calculations envisaged in future studies.

## 3. Models and Methods

### 3.1. Models

The thermodynamics of wetted carbon surfaces is dominated by van der Waals wall/water interactions. In molecular simulations, we model these interactions in terms of the Lennard-Jones potentials *U_ij_* between atoms of types *i* and *j* at the distance *r_ij_*

(2) $$U_{ij}\left(r_{ij}\right)=4\varepsilon_{ij\;}\;\left[{\left(\frac{\sigma_{ij}}{r_{ij}}\right)}^{12}-{\left(\frac{\sigma_{ij}}{r_{ij}}\right)}^{6}\right]$$

where the energy parameter $\varepsilon_{ij\;}$represents the depth of the potential well and $\sigma_{ij}$ the contact distance for specified atoms. Careful parameterizations compatible with selected models of water have been reported in multiple studies. We use the SPC/E model of water [56] and rely on tested parameters for carbon atoms from previous works. For *sp*^2^ carbon atoms, we apply established [35,42] values $\varepsilon_{CC\;}$ = 236 J mol^−1^ and $\sigma_{CC\;}=3.214\;Å$. These values were originally fitted to reproduce the presumed experimental contact angle θ of ~86° on graphite when truncating *all* interactions at the cutoff distance *r*_c_ = 10 Å [42]. Using only a monolayer of carbon, the same approach leads to about 10° higher contact angle on graphene. After the recognition of experimental artifacts from airborne contaminants present at usual ambient conditions, the estimates of measurable contact angles were revised to 65–70° for *pristine* graphite (and around 80° for suspended graphene) free of impurities [31]. While literature reports a broad range of water contact angles on graphene [29,30,31,32,33,57,58,59], the preponderance of experimental and simulation data for suspended graphene falls in the range of 85 ± 10°. By avoiding the truncation of electrostatic interactions, and by increasing the Lennard-Jones cutoff to *r_c_* = 12 Å, our methodology yields contact angles near these predictions (77° for graphite and 83–87° for graphene) using the original [42] parameterization. Parameters for *sp*^3^ carbon atoms, $\varepsilon_{CC\;}$ = 276 J mol^−1^, $\sigma_{CC\;}=3.5\;Å$, as well as those of *sp* atoms, $\varepsilon_{CC\;}$ = 460 J mol^−1^ and $\sigma_{CC\;}=3.5\;Å,$ are taken from the OPLS force field [36,60]. The above values constitute Set 1 of our LJ parameters. In view of the variations in recommended parameters in the literature [61], we estimate the sensitivity of surface wettabilities on the parameterization by comparing the results from Set 1 with those for somewhat stronger interactions (Set 2 in Table 1) based on refs. [42,62]: $\varepsilon_{CC\;}$= 303 J mol^−1^ and $\sigma_{CC\;}=3.214$ Å for *sp*^2^ and $\varepsilon_{CC\;}$ = 369 J mol^−1^ and $\sigma_{CC\;}$= 3.58 Å for *sp*^3^ carbon atoms.

Water–carbon interaction parameters follow from combining the following rules:

(3) $$\varepsilon_{CO}=\sqrt{\varepsilon_{CC\;}\varepsilon_{OO\;}}\;\mathrm{and}\;\sigma_{CO\;}=\frac{\sigma_{CC\;}+\sigma_{OO}}{2}$$

where we use SPC/E water parameters $\varepsilon_{OO}$ = 650 J mol^−1^ and $\sigma_{OO\;}=3.166\;Å$ [56]. Hydrogen atoms feature negligible Lennard-Jones interactions. Water molecules carried partial charges of −0.8476 *e*_o_ and 0.4238 *e*_o_ on oxygen and hydrogen atoms, respectively. Studies of graphane (GA) showed partial atom charges around ±0.05 *e*_o_ to have negligible effect on surface wettability as also applies to tiny atom charges (~±0.01 *e*_o_) in neat carbon allotropes with atoms in distinct hybridization states. These charges were therefore ignored in the present work.

All simulated systems are periodically replicated along lateral (*xy*) directions (parallel to solid/liquid interfaces). Non-electrostatic atom–atom interactions are truncated at the distance *r_c_* = 12 Å and we used Ewald sums [41] with two-dimensional correction [63] for long-range electrostatics.

### 3.2. Simulation Methods

In the majority of simulations, we characterized interfacial thermodynamics using Grand Canonical Monte Carlo (GCMC) simulations of water between carbon walls (Figure 2) in equilibrium with the bulk liquid at vanishing pressure and temperature *T* = 298 K. The simulation code based on the GCMC algorithm of Adams [64,65] has been described in previous works [66,67]. The method requires knowledge about the excess chemical potential $\mu_{ex}\;$of water. The value $\mu_{ex}=-11.88\;kT$ was used as it leads to vanishing pressure (±15 bar) in bulk phase simulations at ambient *T*. As detailed below, GCMC computations employed surface-integrated wall-water potentials *U*(*z*) (shown in Figure 3), suitable for the test-area calculations of interfacial free energies. Low acceptance of molecule additions and removals required GCMC run lengths of ~2 × 10^9^ attempted Monte Carlo moves for every system.

In a fraction of cases, thermodynamic contact angles from GCMC runs are compared with geometric water/surface contact angles determined in all-atom Molecular Dynamics (MD) simulations of cylindrically shaped sessile droplets as detailed in refs. [34,35,37]. MD simulations involving 6–7 × 10^3^ water molecules were performed with a 2-fs time-step and ~6 ns production following a 2 ns equilibration, about an order of magnitude above the droplet relaxation times (Figure 5).

### 3.3. Wetting Free Energy and Contact Angle Calculations

Complementing prevalent simulation approaches to wettability by direct contact angle calculations [34,35,37,42,43,68,69] or thermodynamic integration [33], in this work we employ a variation in the test-area [70,71] method, where we calculate the wetting free energy ∆γ of the substrate

(4) $$∆\gamma =\gamma_{sl}-\gamma_{sv}=-\gamma_{lv}cos\theta \;={\left(\frac{\partial \Omega }{\partial A_{sl}}\right)}_{\mu ,h,T}=-\frac{h}{2}P_{\left|\right|}$$

Above, *h* is the channel width (25 Å in our systems), $\gamma_{ij}$ the interfacial tension between phases *i* and *j*, subscripts *s*, *l*, and *v* denote solid, liquid, and vapor phases and θ contact angle at liquid/solid adhesion strength $\gamma_{lv}$(1 + cosθ). Surface tension $\gamma_{lv}\;$of SPC/E water has been determined [71] at 63.6 mJ m^−2^. The parallel component of the pressure tensor, $P_{\left|\right|}=P_{xx}=P_{yy}$ in a wetted channel is evaluated using the finite difference expression, as follows:

(5) $$\;P_{\left|\right|}=\frac{<N>kT}{V}+\underset{\Delta V_{xy}\to 0}{\lim}\frac{kT\;ln<e^{-\frac{\Delta U_{\left|\right|}}{kT}}>}{\Delta V_{xy}}≅\frac{<N>kT}{V}-\underset{\Delta V_{xy}\to 0}{\lim}<\frac{∆U_{\left|\right|}}{\Delta V_{xy}}>$$

and the thermodynamic contact angle $\theta =cos^{-1}\left(\frac{hP_{\left|\right|}}{2\gamma_{lv}}\right)$. Ω is the grand potential in the channel with volume *V* =$\;A_{sl}h$ and $A_{sl}$ the wetted area. μ is the chemical potential of the liquid corresponding to the bulk aqueous phase at ambient conditions. <*N*> is the average number of confined molecules in the channel determined in GCMC simulations. $\Delta U_{\left|\right|}\;$ is the energy change associated with small area volume increment $\Delta V_{xy}$ in trial moves corresponding to linear scaling of lateral coordinates (*x,y*). The optimal numerical performance of the method has been found for scaling factors 1 ± δ with 10^−6^ ≤δ≤ 10^−5^. Avoiding the inaccuracies associated with positional scaling at nonuniform solids, we model wall/water interactions using laterally averaged *z*-dependent potentials, *U*(*z*), derived by area integration for mean surface densities of wall atoms. Excellent agreement between wetting predictions from full-atom and area-integrated representations has been established in thorough studies for graphene and graphite model systems [34,43] and the generalization to micropatterned surfaces is grounded on manifested interfacial-tension additivity on smooth, molecularly mixed surfaces [72]. Direct comparisons with contact angles from atomistic MD simulations in Table 1 validate the approach for a subset of allotropes considered in this work. Our present formulation of *U*(*z*) differs from the original one [34,43] in explicitly including the cutoff of atom–atom interactions. For monolayer solid surfaces with atom area-density $\varrho_{C}$ and water–carbon plane separation *z* $\leq \;r_{c},$ the integration of wall/water Lenard-Jones interactions truncated at the cutoff distance *r_c_* is carried out as follows:

(6) $$\begin{array}{c} U\left(z\right)=8\pi \varepsilon_{CO}\sigma_{CO}^{2}\varrho_{C}\int_{0}^{\sqrt{r_{c}^{2}-z^{2}}}\left[\frac{{\sigma_{CO}}^{12}}{{\left(r^{2}+z^{2}\right)}^{6}}-\frac{{\sigma_{CO}}^{6}}{{\left(r^{2}+z^{2}\right)}^{3}}\right]rdr\\ =\frac{2}{5}\pi \varepsilon_{CO}\sigma_{CO}^{2}\varrho_{C}\left\{2\left[{\left(\frac{\sigma_{CO}}{z}\right)}^{10}-{\left(\frac{\sigma_{CO}}{r_{c}}\right)}^{10}\right]-5\left[{\left(\frac{\sigma_{CO}}{z}\right)}^{4}-{\left(\frac{\sigma_{CO}}{r_{c}}\right)}^{4}\right]\right\} \end{array}$$

The wall/water interaction potential *U*(*z*) vanishes for z $\geq \;r_{c}$ and there is no discontinuity at *r_c_*. z denotes the distance between the oxygen atom of a water molecule and the plane occupied by carbon atoms, and *r* is the radial distance in lateral direction. The *z*-dependent integration limit of *r* restricts the integration to distances below the radius *r_c_*.

In more general situations, the solid contains distinct atom types (denoted by subscript α, e.g., for carbon atoms in different hybridization states), and these atoms can reside at different lattice planes (numbered by subscript β with β = 0, 1, 2…) along lateral coordinates *x* and *y*. The spacing between consecutive planes is ∆z). In these cases, the surface/water potential equals the sum over all lattice planes and atom types:

(7) $$U\left(z\right)=\frac{2}{5}\pi \sum_{\alpha }\sum_{\beta }\varepsilon_{C_{\alpha }O}\sigma_{C_{\alpha }O}^{2}\varrho_{C_{\alpha }}\left\{2\left[{\left(\frac{\sigma_{C_{\alpha }O}}{z_{\beta }}\right)}^{10}-{\left(\frac{\sigma_{C_{\alpha }O}}{r_{c}}\right)}^{10}\right]-5\left[{\left(\frac{\sigma_{C_{\alpha }O}}{z_{\beta }}\right)}^{4}-{\left(\frac{\sigma_{C_{\alpha }O}}{r_{c}}\right)}^{4}\right]\right\}$$

Above, *z* represents the distance of water oxygen from the nearest plane of carbon atoms (β = 0) and $z_{\beta }$ = z + β∆z is the distance of oxygen from individual plane β. Each term in the sum over β vanishes when $z_{\beta }\geq \;$*r_c_*. For usual lengths *r_c_*, the truncation only causes a detectable change in the attractive term of the integrated potential. Since our simulations use channel widths *h* in excess of 2*r_c_*, only the nearer of the two confining walls contributes to *U*(*z*).

As shown in the discussion of the results, Section 2, stronger *U*(*z*) translates to increased wettability and smaller contact angle. Equation (7) shows the interaction strength between water and the substrate to increase with the number of planes and respective area-densities of atoms in each plane; however, the contribution of added planes decreases with the recess depth β∆z. When the system contains a mixture of carbon atoms in distinct hybridization states α, wetting is facilitated when species with stronger interactions (e.g., *sp*^3^ atoms with bigger $\varepsilon_{C_{\alpha }O}$) reside at the most exposed plane (β=0).

**Figure 7:**
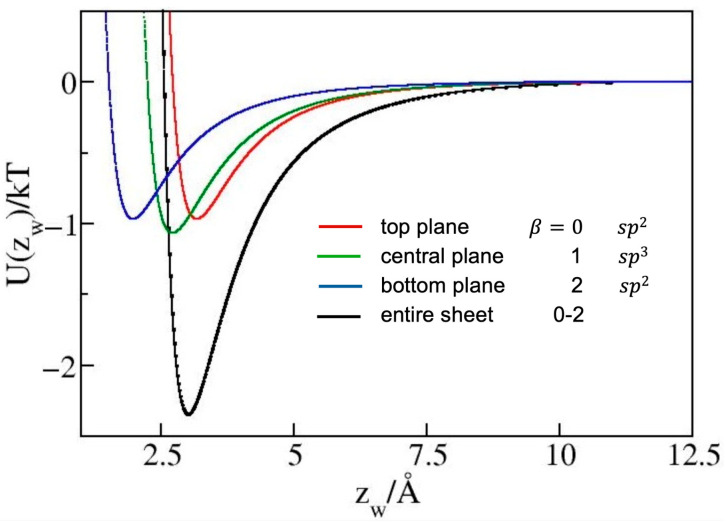
Contributions from separate atomic layers to penta-graphene/water interaction *U*(*z_w_*)/*kT* in a 25 Å wide confinement. Red: contribution from *sp*^2^ atoms in the top lattice plane, green: central plane populated by *sp*^3^ atoms, blue: bottom plane occupied by *sp*^2^ atoms. *z_w_* is the distance from the top lattice plane of C atoms. Of note is the widening of the net attractive well (black), comprising significant contributions from all three levels. Atoms from the top two planes contribute almost equally to the well depth; a slight dominance of the contribution from atoms in the central plane is attributed to a stronger Lennard-Jones interaction of *sp*^3^ carbon atoms.

We consider five types of planar structures. Graphene, a planar carbon allotrope comprised entirely of identical *sp*^2^ carbon atoms [1], as well as ψ-graphene comprising in-plane pentagons and heptagons of *sp*^2^ atoms [10], can be described using Equation (6) with specified densities $\varrho_{C}$. γ-graphyne, a monolayer containing equal proportions of *sp*^2^ and *sp* carbon atoms [21] requires Equation (7) with summation over species α. In the remaining two structures, graphane and penta-graphene, tetrahedral coordination of *sp*^3^ atoms leads to multi-plane configurations. Graphane, a hydrocarbon with a formula (CH)_n_ consists of saturated (*sp*^3^) atoms, each with a single C-H bond and three C-C bonds. The near-tetrahedral coordination is accommodated via alternating atom positioning at ~0.23 Å above and below the central plane [12]. This requires the use of Equation (7) with summation over atomic plane levels β=0 or 1. Consistent with the OPLS force field, the united-atom representation we use in the description of CH units in graphane does not include hydrogen as a separate species. The rationale is provided in [37], where we presented a comparison with a model including hydrogen atoms. For a given force field, the inclusion of hydrogen does not lead to a statistically significant change in contact angle of water on graphane because of negligible LJ interactions of H atoms and very small partial charges on C and H atoms. The three-dimensional structure of penta-graphene involves three lattice planes (β=0, 1 or 2) separated by 0.6 Å, with the central plane occupied by *sp*^3^ atoms while equal populations of *sp*^2^ atoms reside on each of the outside planes prompting the use of Equation (7) with double summation. Figure 7 illustrates the contributions to *U*(*z*) from atoms on distinct atomic planes at the penta-graphene interface.

To validate wettability estimates based on the coarse-grained potential *U*(*z*), (Equations (6) and (7)), in a number of systems we compare predictions from GCMC/test-area calculations for area-averaged *U*(*z*) with sessile-drop contact angles from full-atom MD simulations. For this purpose, we use earlier results for graphene with two different Lennard-Jones parameterizations [34] (Sets 1 and 2), graphene [37] (Set 1), and new simulations of a sessile drop on a full-atom penta-graphene sheet with detailed topology from ref. [15]. In penta-graphene system we use Lennard-Jones parameters from Set 2, which amplify the contrast between *sp*^2^ and *sp*^3^ carbon atoms. This choice can potentially increase the importance of molecular detail, thus providing a more stringent benchmark against which we test the adequacy of the coarse-grained approach to multilevel structures. All MD simulations were carried out with Lammps package (December 2018 version) [73] and concerned sessile drops containing above 6 × 10^3^ molecules. The drops were of hemi-cylindrical shape periodically replicated along the longitudinal axis. Straight three-phase contact lines secured by the cylindrical geometry remove the line tension effects of concern with spherical nanodroplets [74]. The details of MD simulations follow refs. [34,37]. In view of liquid-vapor contour distortions due to the layering of the liquid in the proximity to the substrate, MD contact angles were deduced from circular contour fits of the drops above the height of the second minimum of wall/water distribution function [68], typically ~8Å from the nearest substrate atoms.

## 4. Conclusions

Interfacial free energies estimated by molecular simulations for a representative set of planar carbon allotropes indicate small differences in their wetting behaviors in contrast to notable variations in mechanical and electric properties of these compounds. A rationale for this finding is provided by the small number of distinct building blocks, i.e., carbon atoms with *sp*^2^, *sp*^3^, and *sp* hybridizations (listed in the order of prevalence), comparable van der Waals interaction strengths of *sp*^2^ and *sp*^3^ carbon atoms, and only moderate surface buckling upon the inclusion of tetrahedrally coordinated atoms. Moreover, the known averaging over texture features [72,75] blurs the effects of atomic-scale surface patterning, as manifested by the agreement between the results of full atom and coarse-grained representations. While this work concerns only a subset of known or predicted planar carbon allotropes, it suggests a plausible generalization to new materials in this class, which continue to emerge from first principles calculations. Force field refinements such as the inclusion of polarizability, induction effects, or small partial charges on non-equivalent sheet atoms are not expected [35,37,76,77] to change the qualitative picture, although they may become instrumental in planned extensions to systems with ionic species [78] or polar functionalities necessary to improve the allotropes’ dispersibility in aqueous media.

## Figures and Tables

**Table 1:** Simulation parameters for graphene (G), γ-graphyne (GY), ψ-graphene (ψ-G), penta-graphene (PG), graphane (GA), and graphite (GT) systems with 1–3 atomic layers at distinct lattice planes, the plane’s recess relative to the top lattice plane, carbon hybridization in each layer and associated carbon-carbon Lennard-Jones (LJ) parameters $\varepsilon_{CC\;}$ and $\sigma_{CC,\;}$ area density of carbon atoms in the specified hybridization state in a layer, $\varrho_{C}$, overall and hydration-layer compressibilities, κ and $\kappa_{h}$, and thermodynamic contact angles from GCMC simulations. In selected systems, we also list geometric contact angles from MD simulations in refs. [34,35] (a), ref. [37] (b), and this work (c). Sets 1 and 2 correspond to distinct LJ parameterizations. Statistical uncertainties estimated from block-averaging were about ±3% for compressibilities, ±1–2° for contact angles and ±0.1 mJ m^−2^ for wetting free energies. These estimates exclude systematic errors associated with the choice of model parameters and model simplifications.

Type	Atomic Plane	$\frac{\mathrm{recess}}{Å}$	*sp^n^*	$\frac{\varepsilon_{CC\;}}{J\;{\mathrm{mol}}^{-1}}$	$\frac{\sigma_{CC\;\;}}{Å}$	$\frac{\varrho_{C}}{{Å}^{-2}}$	$\frac{10^{5}\kappa }{{\mathrm{bar}}^{-1}}$	$\frac{10^{5}\kappa_{h}}{{\mathrm{bar}}^{-1}}$	$\frac{\Delta \gamma }{{\mathrm{mJ m}}^{-2}}$	$\theta_{\begin{array}{c} test\;\\ area \end{array}}^{GCMC}$	$\theta_{\begin{array}{c} drop\\ CA \end{array}}^{MD}$
*Set* 1											
G	1st	0	*sp* ^2^	236	3.214	0.38	6.03	9.6	−7.6	83°	87° ^a^
Gy	1st 1st	0 0	*sp* ^2^ *sp*	236 460	3.214 3.58	0.146 0.146	6.88	12.5	−4.5	86°	
ψ-G	1st	0	*sp* ^2^	236	3.214	0.37	6.63	11.7	−6.6	84°	
PG	1st 2nd 3rd	0 0.6 1.2	*sp* ^2^ *sp* ^3^ *sp* ^2^	236 276 236	3.214 3.5 3.214	0.151 0.151 0.151	6.0	9.4	−8.9	82°	
GA	1st 2nd	0 0.46	*sp* ^3^ *sp* ^3^	276 276	3.5 3.5	0.181 0.181	5.5	7.7	−17.5	74°	73° ^b^
GT	1st 2nd 3rd	0 3.4 6.8	*sp* ^2^ *sp* ^2^ *sp* ^2^	236 236 236	3.214 3.214 3.214	0.38 0.38 0.38	5.6	8.0	−14.8	77°	
*Set* 2											
G	1st	0	*sp* ^2^	303	3.214	0.38	5.4	7.4	−17.1	74°	77° ^a^
Gy	1st 1st	0 0	*sp* ^2^ *sp*	303 460	3.214 3.58	0.146 0.146	5.7	8.5	−10.0	81°	
ψ-G	1st	0	*sp* ^2^	303	3.214	0.37	5.55	7.9	−14.9	76°	
PG	1st 2nd 3rd	0 0.6 1.2	*sp* ^2^ *sp* ^3^ *sp* ^2^	3.214 3.58 3.214	303 369 303	0.151 0.151 0.151	5.3	7.0	−17.6	74°	73° ^c^
GA	1st 2nd	0 0.46	*sp* ^3^ *sp* ^3^	369 369	3.58 3.58	0.181 0.181	5.0	6.0	−24.6	67°	
GT	1st 2nd 3rd	0 3.4 6.8	*sp* ^2^ *sp* ^2^ *sp* ^2^	303 303 303	3.214 3.214 3.214	0.38 0.38 0.38	5.2	6.8	−23.0	69°	

## Data Availability

Data are contained within the article.
